# Identification and Transcriptome Analysis of *Bursaphelenchus xylophilus* with Excellent Low Temperature Resistance

**DOI:** 10.3390/ijms252413732

**Published:** 2024-12-23

**Authors:** Yue Zhang, Ruiwen Zhao, Tingting Jing, Sixi Lin, Xiaolei Ding

**Affiliations:** 1 Co-Innovation Centre for Sustainable Forestry in Southern China, Forestry and Grassland, College of Soil and Water Conservation, Nanjing Forestry University, Nanjing 210037, China; zyue@njfu.edu.cn (Y.Z.); raven97@njfu.edu.cn (R.Z.); jingtingting@njfu.edu.cn (T.J.); lsx@njfu.edu.cn (S.L.); 2 Jiangsu Key Laboratory for Prevention and Management of Invasive Species, Nanjing 210037, China

**Keywords:** *Bursaphelenchus xylophilus*, gene expression, temperature resistance

## Abstract

*Bursaphelenchus xylophilus* is one of the most destructive quarantine pests, causing irreversible damage to pine trees. However, the unexpected identification of pine wilt disease in Northern China indicates that *Bursaphelenchus xylophilus* can survive under low temperatures. In this study, we analyzed the reproductivity variations among 18 different isolates, and SC13 was identified to have excellent low temperature resistance. Subsequent molecular analysis of SC13 indicated its distinct gene expression under low temperatures. The epidermal growth factor, nematode cuticle collagen and G-protein-coupled receptor genes with environmental adaptation functions were demonstrated to be differentially expressed under low temperatures. Meanwhile, morphological observations also indicated that SC13 contained significantly more lipid drops in low-temperature treatments. Generally, the identification of representative *Bursaphelenchus xylophilus* isolates will facilitate relevant studies in the future, and the discovery of the gene expression and morphological changes of *Bursaphelenchus xylophilus* under low temperatures could expand the current understanding of the environmental adaption abilities of such invasive nematodes.

## 1. Introduction

Pine wilt disease (PWD) caused by *Bursaphelenchus xylophilus* is the most devastating forest disease in China. *B. xylophilus* has now been found in Asia and Europe, posing a great threat to the local ecological system [1,2]. It has hence been listed as the most dangerous invasive pest by the General Administration of Quality Supervision, Inspection and Quarantine (China) [3]. According to an official announcement from the National Forestry and Grassland Administration of China, this invasive species has currently spread to 18 provinces and 663 counties since its first discovery in 1982. In the past, scholars have proposed that areas with average annual temperatures above 15 °C are suitable for *B. xylophilus* [4], while they cannot survive under low temperatures (<10 °C) [5,6]. Since 2016, a large number of pine trees in Dalian, Fushun and other areas in North China (Liaoning Province) have been affected by *B. xylophilus*. Based on actual data from the China Meteorological Data Service Centre, the annual temperature in Liaoning Province normally remains below 10 °C. Therefore, the unexpected detection of *B. xylophilus* in such areas indicates that this nematode has acquired the ability to surpass the temperature limit and to survive in cold environments. Recently, researchers have also suggested that the disease will continue to spread further in North China [7,8,9]. Meanwhile, other studies have suggested that the *B. xylophilus* infection areas might expand to colder regions in Asia and Europe [10,11,12].

Obviously, the vast and rapid dispersal of *B. xylophilus* indicates its extraordinary ability to deal with various environmental conditions. Relevant studies have suggested that many invasive species could alter their lifespans, morphological characteristics and secondary metabolism to improve their survival rates under extreme environmental conditions [13,14]. Among all environmental factors, the temperature factor is believed to have the most dominant effect on the survival and development of *B. xylophilus* [15,16]. The mechanism of temperature adaptation has been widely investigated in the model nematode *Caenorhabditis elegans* [17,18,19,20]. There are also some studies that have focused on the temperature resistance of *B. xylophilus*. It is reported that metabolite changes and the accumulation of biochemical compounds like fatty acids, carbohydrates and trehalose are associated with the low temperature resistance of *B. xylophilus* [16,21,22]. Additionally, the boosted expression of Cytochrome P450, G-protein-coupled receptors and cyclic guanosine monophosphate genes was found to participate in the molecular regulation of the low temperature adaptation of *B. xylophilus* [23,24,25].

Generally, the aforementioned studies treated *B. xylophilus* with low temperatures to observe the corresponding physiological and molecular changes, without considering the inborn temperature resistance of *B. xylophilus*. Thus, we believe that it would be beneficial to identify potential *B. xylophilus* isolates with excellent low temperature resistance, which would better assist relevant analyses of its temperature resistance.

Therefore, we collected 18 *B. xylophilus* isolates from eight provinces in China. The survival abilities of these nematode strains under various temperatures were measured to identify certain isolates with an extraordinary ability to withstand low temperatures. Subsequent transcriptome sequencing, quantitative PCR and morphological observation were used to further describe the potential mechanism of low temperature resistance in such isolates. The identification and investigation of *B. xylophilus* isolates with superior temperature resistance could provide new perspectives on the temperature resistance mechanisms of such nematodes.

## 2. Results

### 2.1. Reproductivity Variations of B. xylophilus Isolates Under Different Ambient Temperatures

**Figure 1:**
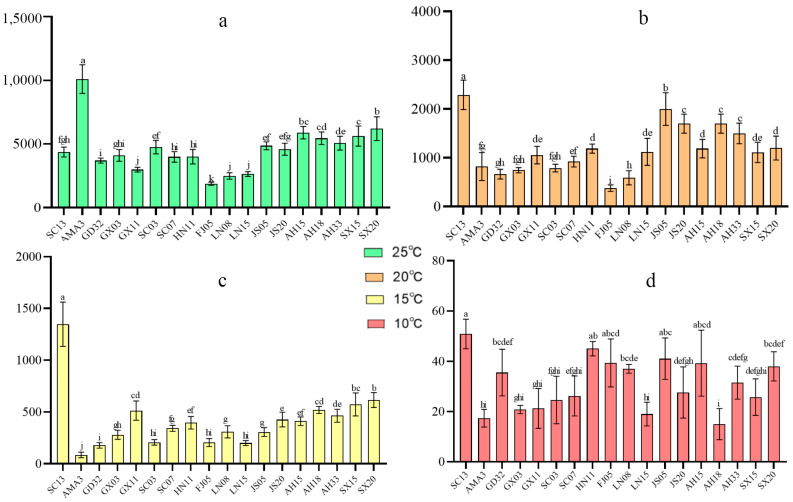
Analysis of reproductivity significance of *B. xylophilus* under various temperature gradients. (**a**) 25 °C, (**b**) 20 °C, (**c**) 15 °C, (**d**) 10 °C. On the bar chart, different letters represent different significance groups. The same letter indicates no significant difference between the two groups, while a different letter indicates a significant difference between the two groups. The use of the same letter in different groups is not significant, as long as the letters are different, it is significant.

The reproductivity of 18 *B. xylophilus* isolates was examined under 10 °C, 15 °C, 20 °C and 25 °C. Generally, the number of *B. xylophilus* individuals decreased as the temperature decreased (Figure 1). Specifically, all *B. xylophilus* isolates showed slight reproductive variations (ranging from ~2000 to ~6000 individuals), except for AMA3, which showed an excellent regeneration ability compared with the others (Figure 1a). When the temperature dropped to 20 °C, the SC13 and JS05 isolates showed better reproductivity compared with the other isolates (Figure 1b). However, only SC13 exhibited extraordinary reproductivity (~1346 individuals) when treated at 15 °C (Figure 1c). When treated under 10 °C, SC13 still showed the best low temperature resistance among all tested isolates. However, the decrease in the population (<initial inoculation amount) indicated that *B. xylophilus* can only survive under low temperatures (10 °C) and cannot reproduce normally (Figure 1d). According to the significance analysis, it could be seen that all nematode isolates had similar reproduction conditions at 25 °C, and SC13 still maintained outstanding reproduction at 20 °C, 15 °C and 10 °C, which was significantly higher than that of the other nematode isolates. It can be inferred that SC13’s reproduction is not affected by low-temperature environments, showing excellent resistance to low temperatures.

### 2.2. Transcriptome Sequencing of SC13 Under Different Temperatures

**Figure 2:**
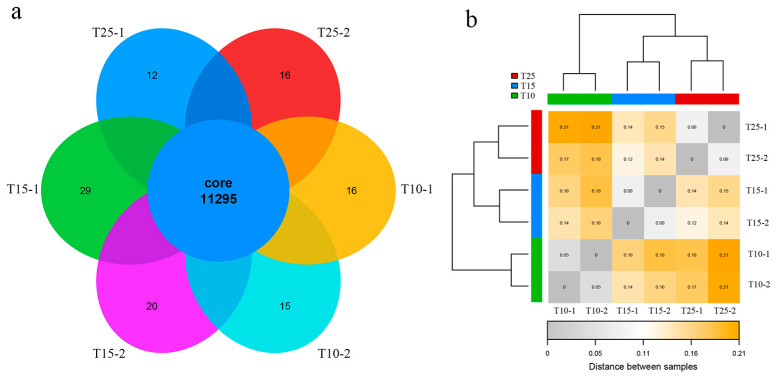
Summary of all identified gene expression patterns in SC13. (**a**) Venn diagram of all expressed genes; (**b**) the sample distance analysis of SC13 under different temperature treatments.

Based on the previous reproductivity test, SC13, with extraordinary low temperature resistance, was used to perform a subsequent RNA-seq analysis (Figure 1). The RNA-seq data of SC13 were generated under 10 °C (T10 treatment), 15 °C (T15 treatment) and 25 °C (T25 control), which was in accordance with the reproductivity test. The average depth of the RNA-seq data was 100X, and approximately 10G bases were obtained for SC13 under different temperatures. Over 96% of the RNA-seq data could align with the *B. xylophilus* genome AH1 (NCBI accession: PRJNA524063, Supplementary Table S1). Moreover, 90% (11295) of the annotated genes were detected across all RNA-seq data, which indicated the high coverage of all gene candidates (Figure 2a). The sample distance analysis based on the overall gene expression indicated that the SC13 isolates under the T15 and T25 treatments were more similar than that of SC13 under T10 (Figure 2b). Overall, the gene expression of SC13 under low temperatures (T15 and T10) showed distinct patterns.

### 2.3. Identification and Functional Annotation of Differentially Expressed Genes in SC13

**Figure 3:**
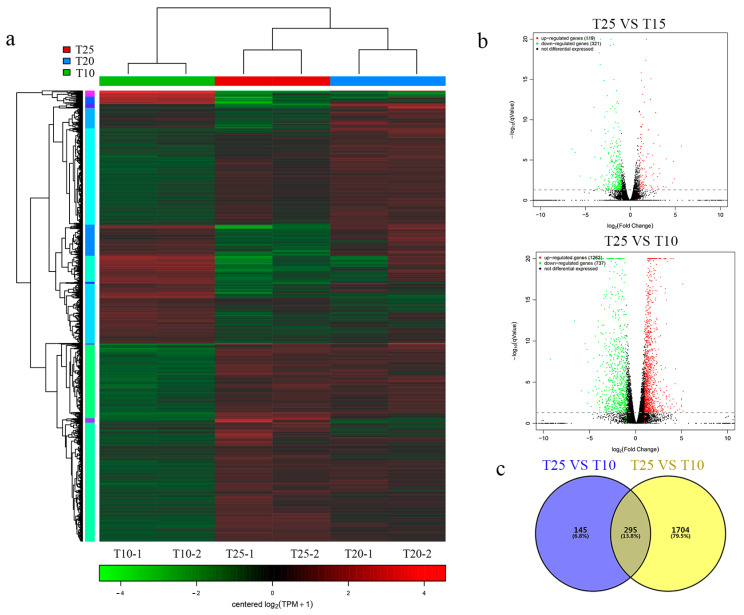
The overall expression pattern of DE genes found in SC13. (**a**) Heatmap illustration of all DE genes (T10-1and T10-2 represent 2 replicates); (**b**) volcano plot of DE genes; (**c**) Venn diagram of all DE genes.

The expression analysis of the differentially (DE) expressed genes further indicated that the T10 treatment yielded an exclusive pattern compared with T25 and T15 (Figure 3a). Specifically, there were 440 and 1999 DE genes in the T15 and T10 treatments compared with T25, respectively. Among all DE genes found in T15, 321 (73%) genes were considered as highly expressed genes, while 119 (27%) were less expressed under the 15 °C treatment. However, there were 737 (37%) highly expressed DE genes in T10, while 1262 (63%) DE genes were less expressed (Figure 3b). Moreover, approximately 295 (13.8%) overlapped DE genes were found across two treatments. Among the above overlapped DE genes, the epidermal growth factor (EGF, gene id: bx1.11657) gene, nematode cuticle collagen (NCC, gene id: bx1.12334) gene and G-protein-coupled receptor gene (GPCR, gene id: bx1.08642) were the top genes (q Value < 0.05) associated with environmental adaptation (Supplementary Table S2). Additionally, the corresponding number of DE genes was significantly increased in the T10 treatment (Figure 3b,c). Therefore, the profiling of the DE genes indicated that dynamic changes in gene expression occurred in SC13 under the low temperature treatments, especially in the T10 treatment.

**Figure 4:**
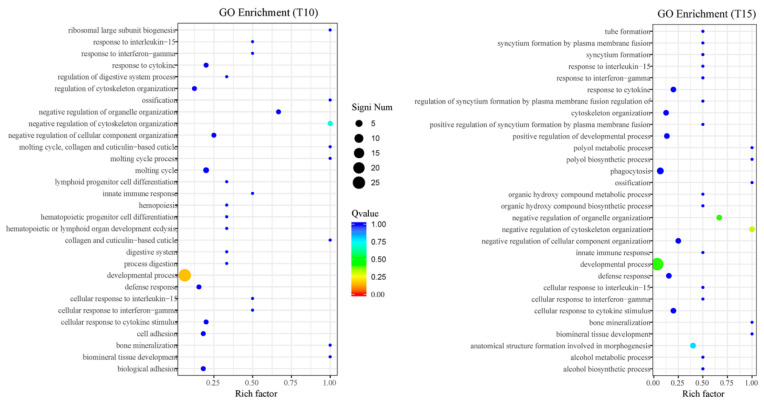
Gene Ontology (GO) enrichment analysis of DE genes in SC13 under T10 and T15.

Moreover, the functional analysis indicated that the DE genes of SC13 under low temperatures were mainly enriched in developmental processes and organelle and cytoskeleton organization (Figure 4). The more significant enrichment of the DE genes in development processes was found in SC13 under the T10 treatment. Notably, significant developmental changes in SC13 were induced by the low temperature treatment.

### 2.4. Experimental Verification of DE Genes and Corresponding Morphological Changes

The AMA3 isolate is widely used in many other *B. xylophilus*-associated studies [26,27]. Here, we also found that AMA3 had prominent reproductivity under T25 but showed poor reproductive ability under low temperatures, which made it suitable to serve as a reference isolate in this study (Figure 1, Supplementary Figure S1). Thus, we further conducted qPCR assays on the aforementioned resistance-associated DE genes of SC13 and AMA3 to verify the possible expression changes of these two isolates.

**Figure 5:**
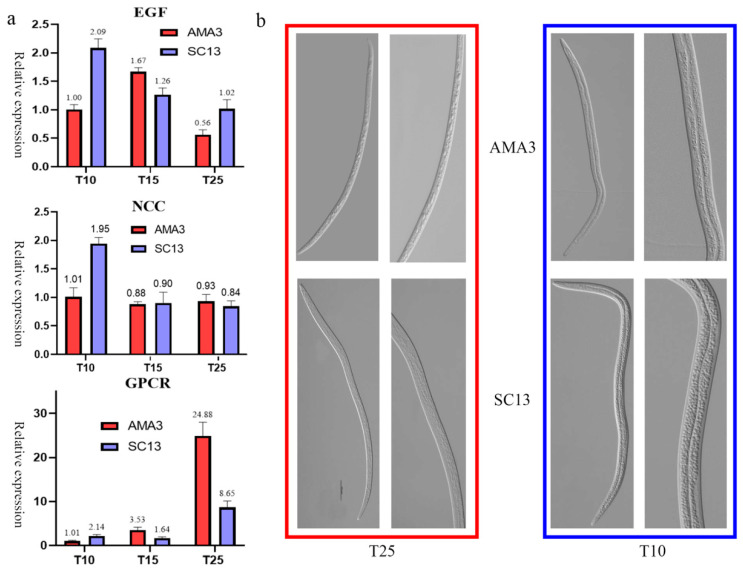
Experimental verification of DE genes and morphological observation of *B. xylophilus*. (**a**) Expression analysis of EGF, NCC and GPCR genes by q-PCR assays; (**b**) morphological changes in AMA3 and SC13 isolates under different temperature treatments.

Generally, the relative expression of the GPCR gene decreased as the temperature dropped in both isolates. Moreover, AMA3 showed significantly higher GPCR expression under T25. Meanwhile, the expression of the NCC gene was stable in both isolates under the different treatments, except for the two-fold boosted expression in SC13 under T10. However, the EGF gene showed higher expression under low temperatures (T15 and T10). Notably, the EGF gene in SC13 was upregulated as the temperature dropped and reached its peak under T10 (Figure 5a).

Meanwhile, the distribution and content of lipid droplets in AMA3 and SC13 were both increased when treated under T10. Morphological observation also showed that SC13 had significantly more lipid droplets compared with AMA3 under T10 (Figure 5b).

## 3. Discussion

The distribution of *B. xylophilus* has expanded to the northern part of China in recent years, which demonstrates that a temperature resistance ability has been gradually acquired during their natural evolution [10,12,28]. To address the invasive risk of *B. xylophilus*, scientists have made many attempts to elucidate the mechanism of such temperature resistance [15,29]. In this study, we aimed to compare the reproductive capacity of 18 *B. xylophilus* isolates and found that some *B. xylophilus* isolates could still reproduce under 15 °C but could only remain alive under 10 °C. Meanwhile, we successfully identified a *B. xylophilus* isolate, SC13, with excellent reproductive and survival abilities under the T15 and T10 treatments, respectively. In other similar studies, scientists have typically treated *B. xylophilus* with low temperatures to observe the corresponding changes, without paying too much attention to the inborn resistance of such nematodes [16,30]. Thus, the identified SC13 could serve as a reference isolate to facilitate temperature adaptation studies in the future.

To further illustrate the prominent resistance mechanism of SC13, we generated RNA-seq data for SC13 under different temperatures according to the previous reproductivity analysis. The overall expression of DE genes was similar between the T25 and T15 treatments, while the T10 treatment showed an exclusive pattern. Moreover, the number of DE genes found in this study was higher than in other relative RNA-seq analyses, indicating more dynamic gene regulation in SC13 under low temperatures [31]. In addition, several genes, like the CYP450, GPCR and cGMP genes, were identified to be closely associated with the temperature response in some similar studies [23,24,25]. Here, we also found that the CYP450 and cGMP genes were differentially expressed, but with low significance.

However, EGF, NCC and GPCR were among the top 10 most significant DE genes across all low temperature treatments, with putative regulatory roles. The GPCR gene has conserved functions related to temperature signal perception and transmission in nematodes [19,32]. The downregulation of the GPCR gene in both AMA3 and SC13 implied a reduction in signal transmission, which may inhibit the low temperature stimulation of *B. xylophilus* [25]. NCC genes are related to cuticle collagen organization and body morphology [33,34,35]. Some studies have indicated that NCC genes were also differentially expressed in *B. xylophilus* when treated with nematicide agents [36,37]. It is reported that the first barrier of nematodes against pathogens, desiccation and other stresses is the cuticle [38,39]. Studies have shown that the high virulence of AMA3 is related to its resistance to external stress. AMA3 also has complete genomic data and transcriptome data, which were generated by many researchers in associated molecular studies [40,41]. It has also been used to investigate the differences in feeding and reproduction of *B. xylophilus* under different environmental conditions, such as the effects of different concentrations of ethanol on the reproduction rate of AMA3 [42]. Therefore, AMA3 and SC13 were selected for the comparative test in this study.

Cuticle collagen genes are stage-specific or cyclically expressed in the epidermis (hypodermis) in model species like *Caenorhabditis elegans* [43]. A change in collagen gene expression is usually induced by environmental stimuli and regulated by transcription factors [44]. Thus, changes in the cuticle collagen genes under low temperatures in *B. xylophilus* may affect the formation of nematode surface tissue and affect the resistance of SC13 to the external environment. The EGF gene has been reported to be associated with prolonging the lifespan of nematodes and regulating organ development in *C. elegans* [45,46,47]. In *B. xylophilus*, EGF was found to be associated with the ability to overcome beta-pinene stress from pine trees [48]. It showed the highest expression in SC13 under the T10 treatment, while the corresponding expression in AMA3 dropped. Such an observation of the EGF gene was not reported in previous studies. Additionally, the GO enrichment analysis in our study also indicated that the most enriched functions involved developmental processes and organelle and cytoskeleton organization. This enrichment result was consistent with our previous observations of lipid droplet changes.

Despite the gene changes, we also found that the content of lipid droplets increased significantly after low temperature treatment in SC13. Although there were some lipid droplets present in AMA3, the overall density was significantly lower than in SC13 (Figure 5b). In previous studies, scientists discovered the formation of lipid droplets in *B. xylophilus* under low temperature treatment and suggested its responsibility for environmental adaptation and longevity extension [15]. In combination, we speculate that the boosted expression of the NCC and EGF genes under low temperature treatment could be responsible for the drastic increase in lipid droplets in SC13. Meanwhile, the reduced expression of the GPCR gene may also impair the interactions of SC13 to deal with adverse environments.

In summary, our study identified a representative strain, SC13, with excellent low temperature tolerance, which could be used for relevant studies to further illuminate the environmental adaptation mechanism of *B. xylophilus.* Meanwhile, the identification of environment-associated genes and morphological changes in SC13 would facilitate an understanding of the low temperature resistance of *B. xylophilus*.

## 4. Materials and Methods

### 4.1. Sampling Information of B. xylophilus

In this experiment, 18 *B. xylophilus* isolates were collated from infected pine trees among 8 provinces across China (Table 1). All isolated *B. xylophilus* were placed on *Botrytis cinerea* medium and cultured under 25 °C to obtain adequate samples for downstream experiments. According to the Baermann funnel method, *B. xylophilus* were isolated from the culture media and stored in sterilized water using 1.5 mL centrifuge tubes.

### 4.2. Reproductivity Test of B. xylophilus Isolates

The water suspension of *B. xylophilus* isolates was manually counted under a Leica DM500 microscope (Leica Microsystems, Shanghai, China)and homogenized to ~100 individuals per 20 μL. The homogenized nematode suspension was injected into a 70 mm PDA plate and cultured in a temperature gradient incubator under 10 °C, 15 °C, 20 °C and 25 °C for 5 days with 5 replicates. Finally, the *B. xylophilus* individuals for every treatment were counted and analyzed with the SPSS version 19.0 software. The morphological observation was carried out with a Zeiss Axio Imager 2 microscope. (ZEISS Vision Care, Shanghai, China).

### 4.3. RNA-Seq Analysis of SC13 Isolate

*B. xylophilus* isolate SC13, cultured under 25 °C, 10 °C and 15 °C, was used to perform the RNA-seq analysis. Total RNA was extracted with Trizol reagent [49] (Thermo Fisher Scientific, Shanghai, China) and sent to the Shanghai Sangon Company (Shanghai, China)to generate RNA-seq data using the Illumina Hiseq™ platform. The raw data were assessed by FastQC (http://www.bioinformatics.babraham.ac.uk/projects/fastqc/) (accessed on 17 October 2023) and filtered with Trimmomatic [31]. The HISAT2 [50] software was used for genome alignment with the AH1 assembly (PRJNA524063). The DeSeq2 software was used to quantify the gene expression and to identify differentially expressed genes [51]. The gene volcano plot and expression heatmap were, respectively, generated by the ggplot2 and gplots packages in R (https://github.com/tidyverse/ggplot2) (accessed on 5 December 2023). The Gene Ontology enrichment analysis was performed with the topGO package (https://www.bioconductor.org/packages/release/bioc/html/topGO.html) (accessed on 5 December 2023).

### 4.4. Quantitative PCR Analysis of Temperature Resistance Gene Candidates

The previously extracted RNAs for transcriptome sequencing were also assessed with the Nanodrop 2000 and 1.2% agarose gel electrophoresis. Qualified RNA was reverse-transcribed into double-stranded cDNA using the Prime Script RT Reagent Kit (Perfect Real Time) (TAKARA, Dalian, China). Specific primers for the qPCR assays were designed with the Primer Premier 6 software (Table 2). The quantitative PCR was conducted with an ABI 7900HT-Sequence Detection System (Applied Biosystems, Carlsbad, CA, USA), with the TB Green Premix Ex Taq II (Tli RNase HPlus) fluorescence quantitative kit (TAKARA, Dalian, China). All qPCR assays were repeated three times and the relative expression was compared with the actin gene.

### 4.5. B. xylophilus Data Measurement

A 20 μL drop of the isolated *B. xylophilus* suspension was covered with a coverslip and placed under a microscope to perform morphology observation under a Zeiss fluorescence microscope (Axioscope 5, ZEISS Vision Care, Shanghai, China). Measurement of the nematodes was performed using the ZEN software (ZEISS ZEN 3.9).

## Figures and Tables

**Table 1:** Sampling information of all *B. xylophilus* isolates.

ID	Sampling Location	Host	Sampling Time
HN11	Shaoyang County, Jiugongqiao Town, Jiugongqiao Village, Guangxi Province	*Pinus massoniana*	29 March 2019
GX03	Shinan Town, Xingye County, Guangxi Zhuang Autonomous Region	*P. massoniana*	12 August 2016
GX11	Shaqi Village, Shatou Town, Cangwu County, Wuzhou, Guangxi Province	unknown	24 March 2019
SC03	Jinchong, Zhou’an Village, Fushan Town, Fushun County, Zigong City, Sichuan Province	*P. massoniana*	20 March 2015
SC07	Dachong, Wuli Village, Shuangyi Town, Yibin County, Yibin City, Sichuan Province	*P. massoniana*	30 March 2015
SC13	Yibin City, Sichuan Province	*P. massoniana*	November 2015
FJ05	Fengze District, Quanzhou City, Fujian Province	*P. massoniana*	December 2014
LN04	Dalian, Liaoning Province	unknown	20 October 2016
LN08	Dongling, Shenyang, Liaoning Province	*Pinus tabuliformis*	20 August 2017
LN15	Hongtoushan, Qingyuan County, Fushun, Liaoning Province	*Larix gmelinii*	25 September 2018
JS05	Sun Yat-sen Mausoleum in Nanjing, Jiangsu Province	*P. massoniana*	December 2014
JS20	Changshu City, Jiangsu Province, Yushan Forest Farm Lost Property Stream Protection Shed	*P. massoniana*	11 October 2017
AH15	Huangshan City, Anhui Province Huangshan District Town Taiping Lake Township (forest) Nan’an Village	*P. massoniana*	18 October 2016
AH18	Back hill of Hu Jia house, Zhangcun, Jiaocun Town, Anhui Province	*P. massoniana*	26 July 2017
AH33	Huangshan North Gate Area, Anhui Province	*P. massoniana*	31 October 2018
SX15	Shaping Wusi Village, Dahe Ba Town, Foping County, Hanzhong City, Shaanxi Province	*P. massoniana*	13 August 2017
SX20	Ningshan County, Shanxi Province	*P. tabuliformis*	19 August 2017
AMA3	Anhui Province	*Pinus thunbergii*	10 July 2004

**Table 2:** Primer sequences for qPCR assay.

Gene	Primer ID	Primer Sequence
ACTIN	ACTIN-F	GCAACACGGAGTTCGTTGTA
	ACTIN-R	GTATCGTCACCAACTGGGAT
Epidermal growth factor	QEGF-F	GTGCCGGGACATCAATTC
	QEGF-R	GCCTCAAGCTTTTGTCAGC
G-protein-coupled receptor	QGPCR-F	GCATACACACAGCCTGTAGTC
	QGPCR-R	GAGTGCCAAGCCTTGAGGTAG
Nematode cuticle collagen	QNCC-F	CGGGAGTGCTCTTGTCATTG
	QNCC-R	GGAACTCCACCATCTCCACC

## Data Availability

Strains and primers are available upon request. The authors affirm that all data necessary to confirm the conclusions of the article are present within the article, figures and tables.

## Supplementary Materials

The following supporting information can be downloaded at: https://www.mdpi.com/article/10.3390/ijms252413732/s1.

## References

[1] Abelleira A., Picoaga A., Mansilla J.P., Aguin O. (2011). Detection of *Bursaphelenchus xylophilus*, Causal Agent of Pine Wilt Disease on *Pinus pinaster* in Northwestern Spain. Plant Dis..

[2] Robinet C., Roques A., Pan H.Y., Fang G.F., Ye J.R., Zhang Y.Z., Sun J. (2009). Role of human-mediated dispersal in the spread of the pine wood nematode in China. PLoS ONE.

[3] Ding X.L., Ye J.R., Wu X., Huang L., Zhu L.H., Lin S.X. (2015). Deep sequencing analyses of pine wood nematode *Bursaphelenchus xylophilus* microRNAs reveal distinct miRNA expression patterns during the pathological process of pine wilt disease. Gene.

[4] Mamiya Y. (1983). Pathology of the Pine Wilt Disease Caused by *Bursaphelenchus xylophilus*. Annu. Rev. Phytopathol..

[5] Zheng Y.N., Liu P.X., Shi Y., Wu H., Yu H.Y., Jiang S.W. (2021). Difference analysis on pine wilt disease between Liaoning Province of northeastern China and other epidemic areas in China. J. Beijing For. Univ..

[6] Ohsawa M., Akiba M. (2014). Possible altitude and temperature limits on pine wilt disease: The reproduction of vector sawyer beetles (*Monochamus alternatus*), survival of causal nematode (*Bursaphelenchus xylophilus*), and occurrence of damage caused by the disease. Eur. J. For. Res..

[7] Ye J.R., Wu X.Q. (2022). Research progress on Pine Wilt Disease. Chin. For. Pests Dis..

[8] Ye J.R. (2019). Epidemic Status of Pine Wilt Disease in China and Its Prevention and Control Techniques and Counter Measures. Sci. Silvae Sin..

[9] Zhao H.X., Xian X.Q., Yang N.W., Guo J.Y., Zhao L.L., Sun J.H., Shi J., Liu W.X. (2024). Continuum of global to local dispersal frameworks highlights the increasing threat of pine wilt disease in China. Glob. Ecol. Conserv..

[10] Wu H.Y., Tan Q.Q., Jiang S.X. (2013). First Report of Pine Wilt Disease Caused by *Bursaphelenchus xylophilus* on *Pinus thunbergii* in the Inland City of Zibo, Shandong, China. Plant Dis..

[11] Gruffudd H.R., Jenkins T.A.R., Evans H.F. (2016). Using an evapo-transpiration model (ETpN) to predict the risk and expression of symptoms of pine wilt disease (PWD) across Europe. Biol. Invasions.

[12] Lu Q., Wang W.D., Liang J., Yan D.H., Jia X.Z., Zhang X.Y. (2005). Potential suitability assessment of *Bursaphelenchus xylophilus* in China. Forest Research.

[13] Panov V., Krylov P., Riccardi N. (2004). Role of diapause in dispersal and invasion success by aquatic invertebrates. J. Limnol..

[14] Andreadis S.S., Athanassiou C.G. (2017). A review of insect cold hardiness and its potential in stored product insect control. Crop Prot..

[15] Zhao L.L., Wei W., Kulhavy D.L., Zhang X.Y., Sun J.H. (2007). Low temperature induces two growth-arrested stages and change of secondary metabolites in *Bursaphelenchus xylophilus*. Nematology.

[16] Liu Z., Li Y., Pan L., Meng F., Zhang X. (2019). Cold adaptive potential of pine wood nematodes overwintering in plant hosts. Biol. Open.

[17] Xiao R., Zhang B., Dong Y., Gong J., Xu T., Liu J., Xu X.Z. (2013). A genetic program promotes *C. elegans* longevity at cold temperatures via a thermosensitive TRP channel. Cell.

[18] Kuhara A., Okumura M., Kimata T., Tanizawa Y., Takano R., Kimura K.D., Inada H., Matsumoto K., Mori I. (2008). Temperature Sensing by an Olfactory Neuron in a Circuit Controlling Behavior of *C. elegans*. Science.

[19] Ohta A., Ujisawa T., Sonoda S., Kuhara A. (2014). Light and pheromone-sensing neurons regulates cold habituation through insulin signalling in *Caenorhabditis elegans*. Nat. Commun..

[20] Murray P., Hayward S.A.L., Govan G.G., Gracey A.Y., Cossins A.R. (2007). An explicit test of the phospholipid saturation hypothesis of acquired cold tolerance in *Caenorhabditis elegans*. Proc. Natl. Acad. Sci. USA.

[21] Zhao D., Zheng C., Shi F., Xu Y., Zong S., Tao J. (2021). Expression analysis of genes related to cold tolerance in *Dendroctonus valens*. PeerJ.

[22] Chen Q.L., Zhang R.Z., Li D.L., Wang F., Jiang S.W., Wang J.N. (2021). Trehalose in pine wood nematode participates in DJ3 formation and confers resistance to low-temperature stress. BMC Genom..

[23] Wang B.W., Hao X., Xu J.Y., Wang B.Y., Ma W., Liu X.F., Ma L. (2020). Cytochrome P450 metabolism mediates low-temperature resistance in pinewood nematode. FEBS Open Bio..

[24] Wang B., Ma L., Wang F., Wang B., Hao X., Xu J., Ma Y. (2017). Low Temperature Extends the Lifespan of *Bursaphelenchus xylophilus* through the cGMP Pathway. Int. J. Mol. Sci..

[25] Wang B., Hao X., Xu J., Ma Y., Ma L. (2019). Transcriptome-Based Analysis Reveals a Crucial Role of BxGPCR17454 in Low Temperature Response of Pine Wood Nematode (*Bursaphelenchus xylophilus*). Int. J. Mol. Sci..

[26] Hu L.-J., Wu X.-Q., Ding X.-L., Ye J.-R. (2021). Comparative transcriptomic analysis of candidate effectors to explore the infection and survival strategy of *Bursaphelenchus xylophilus* during different interaction stages with pine trees. BMC Plant Biol..

[27] Qiu Y.J., Wu X.Q., Wen T.Y., Hu L.J., Rui L., Zhang Y., Ye J.R. (2023). The *Bursaphelenchus xylophilus* candidate effector BxLip-3 targets the class I chitinases to suppress immunity in pine. Mol. Plant. Pathol..

[28] Pan L., Cui R., Li Y.X., Feng Y.Q., Zhang X.Y. (2020). Investigation of Pinewood Nematodes in *Pinus tabuliformis* Carr. under Low-Temperature Conditions in Fushun, China. Forests.

[29] Pan L., Cui R., Li Y.X., Zhang W., Bai J.W., Li J.W., Zhang X.Y. (2021). Third-Stage dispersal juveniles of *Bursaphelenchus xylophilus* can resist low-temperature stress by entering cryptobiosis. Biology.

[30] Li Z., Tao J., Zong S. (2022). Cold Tolerance in Pinewood Nematode *Bursaphelenchus xylophilus* Promoted Multiple Invasion Events in Mid-Temperate Zone of China. Forests.

[31] Bolger A.M., Lohse M., Usadel B. (2014). Trimmomatic: A flexible trimmer for Illumina sequence data. Bioinformatics.

[32] Ohta A., Kuhara A. (2013). Molecular mechanism for trimetric G protein-coupled thermosensation and synaptic regulation in the temperature response circuit of *Caenorhabditis elegans*. Neurosci. Res..

[33] Fernando T., Flibotte S., Xiong S., Yin J.H., Yzeiraj L.R., Moerman D.G., Melendez A., Savage-Dunn C. (2011). *C. elegans* ADAMTS ADT-2 regulates body size by modulating TGF beta signaling and cuticle collagen organization. Dev. Biol..

[34] Banerjee S., Gill S.S., Jain P.K., Sirohi A. (2017). Isolation, cloning, and characterization of a cuticle collagen gene, Mi-col-5, in *Meloidogyne incognita*. 3 Biotech.

[35] Madaan U., Yzeiraj E., Meade M., Clark J.F., Rushlow C.A., Savage-Dunn C. (2018). BMP signaling determines body size via transcriptional regulation of collagen genes in *Caenorhabditis elegans*. Genetics.

[36] Chen Y., Zhou X., Guo K., Chen S.N., Su X. (2021). Transcriptomic insights into the effects of CytCo, a novel nematotoxic protein, on the pine wood nematode *Bursaphelenchus xylophilus*. BMC Genom..

[37] Chen J., Hao X., Wang B.Y., Ma L. (2022). Transcriptomics and coexpression network profiling of the effects of levamisole hydrochloride on *Bursaphelenchus xylophilus*. Pestic. Biochem. Phys..

[38] Gravato-Nobre M.J., Vaz F., Filipe S., Chalmers R., Hodgkin J. (2016). The invertebrate lysozyme effector ILYS-3 is systemically activated in response to danger signals and confers antimicrobial protection in response to danger signals and confers antimicrobial protection in *C. elegans*. PLoS Pathog..

[39] Xiong H.J., Pears C., Woollard A. (2017). An enhanced *C. elegans* based platform for toxicity assessment. Sci. Rep..

[40] Rui L., Liu H., Liang R., Wu X. (2021). Resistance genes mediate differential resistance to pine defensive substances α-Pinene and H_2_O_2_ in *Bursaphelenchus xylophilus* with different levels of virulence. J. For. Res..

[41] Ding X., Guo Y., Ye J., Wu X., Lin S., Chen F., Zhu L., Huang L., Song X., Zhang Y. (2022). Population differentiation and epidemic tracking of *Bursaphelenchus xylophilus* in China based on chromosome-level assembly and whole-genome sequencing data. Pest Manag. Sci..

[42] Wang M., Wang L.S., Fang J.N., Du G.C., Zhang T.T., Li R.G. (2022). Transcriptomic profiling of *Bursaphelenchus xylophilus* reveals differentially expressed genes in response to ethanol. Mol. Biochem. Parasitol..

[43] Yin J.H., Madaan U., Park A., Aftab N., Savage-Dunn C. (2015). Multiple cis elements and GATA factors regulate a cuticle collagen gene in *Caenorhabditis elegans*. Genesis.

[44] Mesbahi H., Pho K.B., Tench A.J., Guerrero V.L.L., MacNeil L.T. (2020). Cuticle collagen expression is regulated in response to environmental stimuli by the GATA transcription factor ELT-3 in *Caenorhabditis elegans*. Genetics.

[45] Detienne G., De Haes W., Ernst U.R., Schoofs L., Temmerman L. (2014). Royalactin extends lifespan of *Caenorhabditis elegans* through epidermal growth factor signaling. Exp. Gerontol..

[46] Hill A.J., Mansfield R., Lopez J., Raizen D.M., Van Buskirk C. (2014). Cellular Stress Induces a Protective Sleep-like State in *C. elegans*. Curr. Biol..

[47] Mereu L., Morf M.K., Spiri S., Gutierrez P., Escobar-Restrepo J.M., Daube M., Walser M., Hajnal A. (2020). Polarized epidermal growth factor secretion ensures robust vulval cell fate specification in *Caenorhabditis elegans*. Development.

[48] Li Y.X., Feng Y.Q., Wang X., Cui J., Deng X., Zhang X.Y. (2020). Adaptation of pine wood nematode *Bursaphelenchus xylophilus* to β-pinene stress. BMC Genom..

[49] Ding X., Ye J., Lin S., Wu X., Li D., Nian B. (2016). Deciphering the Molecular Variations of Pine Wood Nematode *Bursaphelenchus xylophilus* with Different Virulence. PLoS ONE.

[50] Kim D., Paggi J.M., Park C., Bennett C., Salzberg S.L. (2019). Graph-based genome alignment and genotyping with HISAT2 and HISAT-genotype. Nat. Biotechnol..

[51] Love M.I., Huber W., Anders S. (2014). Moderated estimation of fold change and dispersion for RNA-seq data with DESeq2. Genome Biol..

