# Supplementary material for: Identification and Transcriptome Analysis of Bursaphelenchus xylophilus with Excellent Low Temperature Resistance
Source: Int J Mol Sci. 2024 Dec 23;25(24):13732. doi: 10.3390/ijms252413732 (PMC11679782; doi:10.3390/ijms252413732)
Supplement: Supplementary file 1 [file ijms-25-13732-s001.zip › Supplementaty Figures.pdf]

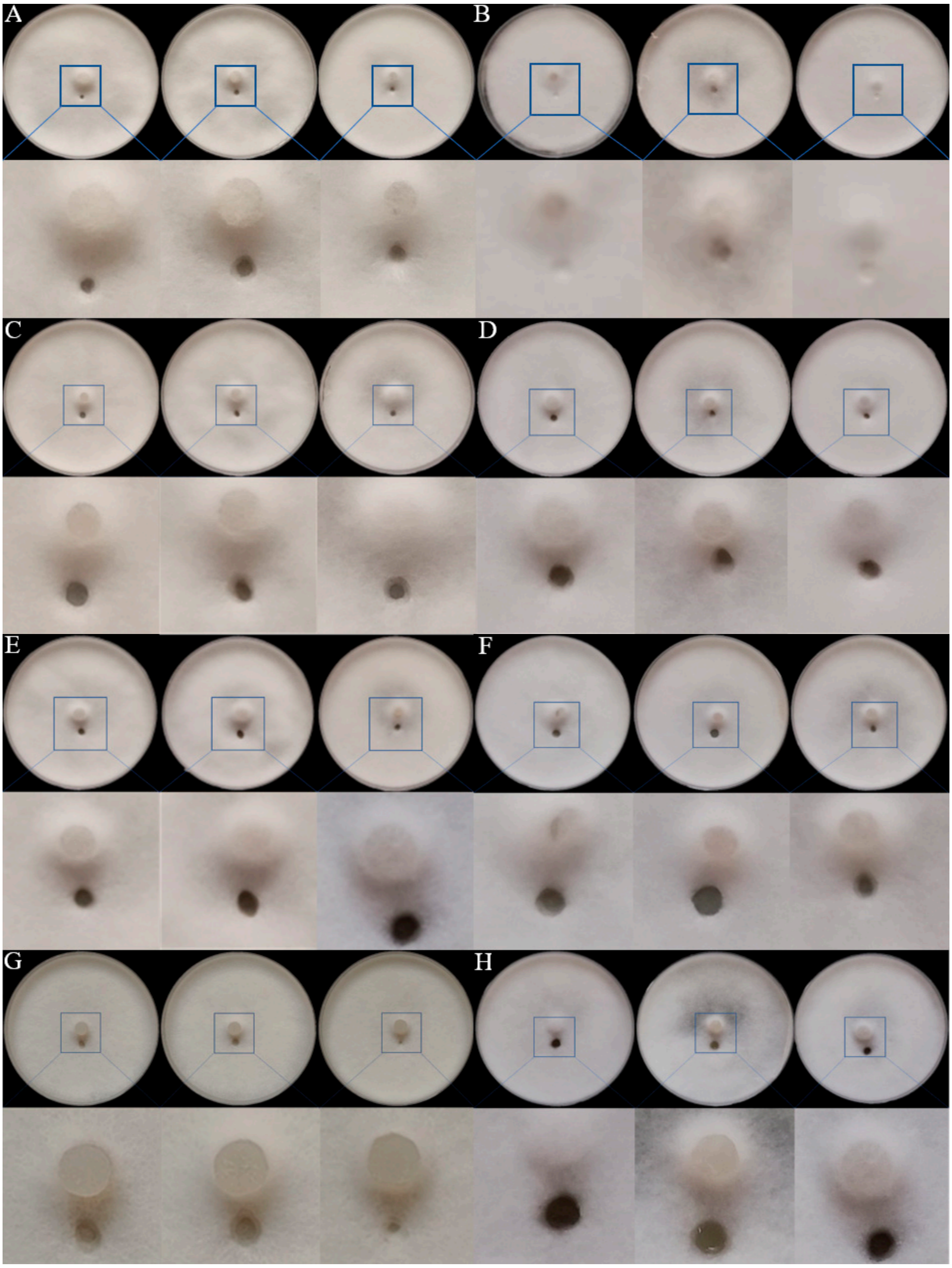

Supplementary Figure S1. Feeding status of *B. xylophilus* after 5 days of inoculation on *B. cinerea* (Blue boxes indicated the feeding holes on *B. cinerea*, the size of the holes represented the consuming speed of *B. xylophilus*)

A: SC13 10℃ C: SC13 15℃ E: SC13 20℃ G: SC13 25℃

B: AMA3 10℃ D: AMA3 15℃ F:AMA3 20℃ H: AMA3 25℃
